# Odontalgia

**Published:** 1840-05

**Authors:** S. P. Hullihen

**Affiliations:** Wheeling, Va.


### ODONTALGIA.

*Observations on Tooth-ache, by* S. P. Hullihen, *of Wheeling, Va.*

There is no disease, perhaps, which attacks the human family so indiscriminately and so universally, as the tooth-ache; none which the dentist is called upon so often to cure, and none that he treats with less success.

There can be few things in surgery more erroneous than to call every painful affection of the teeth "tooth-ache," and to treat them all as one specific disease. As well might a wound in the scalp, a fracture of the skull, or inflammation of the brain, be called "head-ache," and treated as such.

Tooth-ache most generally originates from local causes, differing sometimes materially from each other. However, pains are oft-times experienced in the teeth, through that mysterious agent, usually termed sympathy: when the exciting cause may exist in a distant part of the system. Females are peculiarly liable to be affected in this way during pregnancy.

The pain of a tooth is always annoying, and sometimes exceedingly severe; but this is trifling, compared with consequences that frequently ensue from it. Diseases of the most malignant nature, and deformities the most unseemly to be beheld, are not unfrequently the results of tooth-ache, or rather the diseases which occasion it.

With these remarks, I shall proceed to treat, separately, of the different causes of tooth-ache, in the following order:—

1st. Tooth-ache from exposure of the nerve.
2nd. From fungus of the nerve.
3d. From the confinement of pus in the internal cavity of a tooth.
4th. From a diseased state of the periosteum covering the fang.
5th. From sympathy.

### *Tooth-ache from Exposure of the Nerve.*

The nerve of a tooth, is intended, by nature, to be protected by a thick bony covering, and when exposed either by accident or disease, it becomes extremely painful from the action of air, food, fluids, and other irritating causes.

The pain is of a sharp throbbing character, and always more or less severe, in proportion to the amount of injury or irritation, that the nerve has received. The pain is generally very easily palliated, but it will immediately return upon the slightest irritation of the nerve. The nerve will sometimes remain troublesome for several months, in other cases it will frequently suppurate upon the first or second severe paroxysm of tooth-ache. But in all cases where the nerve of a tooth becomes fairly exposed, it will sooner or later invariably suppurate, leaving the crown in a state of total necrosis.

Treatment:—The treatment of this kind of tooth-ache may be divided into the palliative and the radical. The palliative, consists in benumbing the sensibility of the nerve; the radical in destroying the nerve.

### *To benumb the sensibility of the nerve.*

The essential oils, opium, kreosote, and various other stimulating remedies have been used, and with more or less success, when actually applied to an exposed nerve. But from the very prevalent opinion that *all* pains of the teeth proceed from an exposure of the nerve, these remedies are often employed, when it is not possible in the nature of things that they should prove in the least beneficial.

The best perhaps of all palliative remedies is a mixture of morphia and kreosote, made into a thin paste. This preparation may be applied to the nerve on a small pledget of lint, and will most generally insure immediate relief.

But in order to be generally successful in destroying the nerve of a tooth, it is necessary to resort to different means in different cases.

When the crown of a tooth is excised or broken off, leaving an exposed nerve, there is no means of destroying it so efficiently as the actual cautery. The instrument should be highly heated and plunged quickly into the nerve down the whole length of the fang, and withdrawed the same instant. The nerve is thereby disorganized, and destroyed instantaneously, producing but little or no pain. I am aware that much has been said against the use and practicability of the actual cautery in destroying the nerve ; that it has been denounced as " unscientific and barbarous in the extreme ;" but I must confess that I am unable to view it in such a light, and therefore beg leave to differ in this point, from many of the most scientific and respectable dentists.

But when the crown of a tooth is to be preserved, and the nerve is exposed, the use of the cautery is generally impracticable. But in all such cases where the tooth has but one fang, the nerve may be crushed. This is done by taking a small wire or splinter of hickory, so shaped, as to correspond with the internal cavity of the tooth, and plunging it into the nerve, down the entire length of the fang. By this operation the structure of the nerve is completely broken up and destroyed. But when a tooth has two or more fangs, neither the cautery nor the crushing of the nerve can be resorted to with success. The nerves of such teeth should be suffered to remain unmolested until they suppurate ; a process, by which nature sooner or later invariably removes every exposed nerve. With this view, the nerve should be covered with a thin plate of gold or silver, in such a manner as to prevent the least possible pressure on it, and the tooth should then be plugged. A tooth treated in this manner will sometimes remain free from pain for several months, but at length it will become a little painful and appear longer than the rest, and finally sore to the touch ; symptoms that indicate that the nerve is suppurating. The plug should now be immediately removed and the pus suffered to escape through the opening in the crown of the tooth. If this be neglected an alveolar abscess will form. Of this I will treat at length, when I come to speak of tooth-ache from the confinement of pus in the internal cavity of a tooth.

### *Tooth-ache from fungus of the nerve.*

Fungus of the nerve, so called from its occupying the natural cavity of the tooth, most probably originates from a remnant of the dental blood vessels. It always attacks teeth in which the nerve has suppurated, and is of a deep red color ; very soft ; bleeds freely upon the slightest touch ; is sometimes totally insensible, at others, highly sensitive, giving much pain of an incessant character ; entirely free from any darting or throbbing sensation ; and when small and painful, is frequently mistaken for an exposed nerve. It varies in size, from that of a pin's head, to a pea, and larger. It is sometimes, when small, deeply situated in a fang ; but more generally protrudes, filling up a cavity formed by caries.

Treatment.—To destroy this diseased growth, is very difficult, and I may say, impossible, except in such cases, as permit the actual cautery to be applied to its root; and, as the disease commences in the very foramen of the internal cavity of the fang, this means is narrowed down to very few cases when at all practicable. When a tooth becomes painful from this affection, it is only necessary to make the fungus bleed freely, to ensure immediate relief; but in all cases, where the growth cannot be completely eradicated, the tooth should be extracted, for there is no disease of the mouth, which makes the breath so intolerable.

Some time since, a young lady, about seventeen years of age, applied to me with a growth of this kind, in each of the second molares of the under jaw, which had assumed rather a novel character. She stated that the fungi had made their appearance in both teeth at the same time, about four years before, and that for the last two years she had been much troubled with a bleeding from them, which occurred regularly once a month, and continued several days. She being anxious to have the teeth saved, I destroyed, to all appearance, the morbid growth, and plugged the teeth. In a few days they became sore and painful; the plugs were removed, and a slight bleeding commenced, which continued for three or four days, and then the tumours entirely disappeared. I was therefore induced to plug them again; but in about three weeks, the teeth became sore, the plugs were removed, and a bleeding ensued as before. I now suspected it to be a *vicarious menstruation*, which it proved to be, and mentioned the case to the family physician. At his request, I plugged them again, and the result was precisely the same as before. The teeth were then removed, and the patient was put under a course of treatment by her physician, which effected a cure.

### *Tooth-ache from the confinement of pus in the internal cavity of a tooth.*

Whenever the nerve of a tooth is rendered near the external surface, by caries, fractures, or from the absorption and receding of the alveolar process and gum, or by other causes, it is subject to much irritation, varying however in proportion to the extent and loss of covering.

Thus a greater or lesser inflammation of the nerve, neessarily ensues, producing the following very different effects. When the inflammation is slight, and comes on gradually, some dormant vessels are stimulated to action. A bony deposite takes place in the internal cavity of the tooth, and the nerve is again completely protected from all external irritation. But in a great majority of cases, inflammation is induced more rapidly and runs sooner or later to excess. Suppuration of the nerve is the consequence, and from this result may be traced the true cause of many diseases of the jaws and mouth, not generally understood.

Tooth-ache, from the confinement of pus in the internal cavity of a tooth, occurs more frequently, than any other variety, except from exposure of the nerve. At first the tooth is painful, only when hot or cold fluids pass over it, and may continue in this situation for some time. At length, a steady gnawing pain supervenes, and then the tooth begins to get sore, and appears a little loose and longer than the rest. About this period the pain assumes a very different character, darting from the tooth along the courses of the nerves, to the temple, ear, and side of the head, and to the teeth of both jaws on that side. Within twenty-four hours, the face begins to swell, and the pain changes to one of a constant throbbing description; symptoms which indicate that an alveolar abscess is forming.

When a tooth, in the manner described, becomes painful, the nerve is about suppurating; when it appears longer than the rest, loose, and is very sore, the nerve has suppurated, and the pus is beginning to ooze out at the extremity of the fang, where the vessels enter, producing much pressure, hence the sharp darting paroxysms. When the cheek begins to swell, the matter is effusing between the alveolus and its lining membrane, and occasions the throbbing pain, which always accompanies the formation of an alveolar abscess. The true abscess of the antrum maxillare, originates in like manner, from the same cause, when the fangs are very near or penetrate into that cavity.

There is now and then a case that differs materially from the general progress of this disease. The nerve may suppurate, and the face swell slightly, and both pain and swelling subside, without the development of an abscess. When this occurs, the matter is insinuated between the end of the fang, and the membrane covering it, forming a sac about the size of a pea, and sometimes larger, which may be felt distinctly in the gum, opposite the end of the fang. By pressing on it, a fullness, or sensation of pressure is felt in the tooth. But in time this sac will burst, and an alveolar abscess will be the final result.

Treatment.—As soon as it is ascertained that the nerve is suppurating, the tooth should be trepaned, or drilled. The matter is thereby suffered to escape as soon as formed, and the patient is immediately relieved from pain. In such cases, when the matter has even begun to ooze out at the end of the fang, and the face has not yet become swollen, the same treatment should be observed, and will most generally prove successful.—But when the face is already swollen, and the pain is of a throbbing character, there is no remedy but the extraction of the tooth.

*Tooth-ache from a diseased state of the Periosteum covering the fang.*

This disease is an inflammation and thickening of the periosteum at the point of the fang, causing the formation of pus, and is entirely confined to teeth in which the nerve has suppurated, and produced an alveolar abscess. It is true, the periosteum at the point of the fang becomes slightly inflamed, during a severe inflammation of the nerve, but suppuration is never the result of such inflammation, independently of the nerve, and never occurs until after the nerve has first suppurated, causing an alveolar abscess. The suppuration of the nerve, and the formation of an abscess is always the primary cause of this disease. The pain is generally of a dull, heavy character; the tooth becomes a little sore, the gums very much inflamed, and of a bluish tinge, immediately over and along the whole length of the fang; sometimes the gum is puffy opposite the end of the fang, and the pain of a throbbing description. In such cases, matter is forming, and a partial filling up of an old alveolar abscess takes place, forming, what is usually termed, a "gum boil." It is rarely, if ever, that the face becomes swollen from an attack of this kind of tooth-ache.

Treatment.—The pain may be greatly palliated, by making an incision through the gum, along the entire length of the fang, and then applying a roasted fig, or bruised raisins to the gum; but as the pain is almost sure to return upon taking cold, the extraction of the tooth should always constitute the treatment of this variety of tooth-ache.

### *Tooth-ache from sympathy.*

It is well known that the human system is sympathetically connected, in all its parts, by the nervous system; and yet some organs from being more intimately connected than others, sympathize together more frequently.

During the formation of an alveolar abscess, there is much pain and inflammation in the offending tooth and surrounding parts; the nerves become involved and irritated; the irritation extends to all the teeth of both jaws on that side, and to the ear, temple and scalp. Sometimes each tooth sympathizes in its turn, so that some one is always more severely affected for a short time than the rest, and this more pungent sensation, is continually changing from one tooth to another, and to other associated parts.

Sympathetic tooth-ache occurs very frequently during pregnancy, and is evidently induced, sometimes from an increased, at other times from a diminished action, dependent on the peculiar state of the general system. But in either case the teeth are almost invariably diseased, and the painful sensation, exclusively confined to them. Sufficient indications however, are always present, and so distinctly marked, as to enable the practitioner at once to determine whether the pain proceeds from torpor or an inflammatory action.

Treatment.—When tooth-ache is induced by direct sympathy with a diseased tooth, or the results arising from one, the cause, if possible, should be removed; but if the exciting cause exists in a distant part of the system, blood-letting, and purgatives will sometimes be indicated, and when so, may be recommended. But when a painful sensation is produced from want of action, or natural stimulus, the teeth *should not be extracted*, unless, when so much decayed as to render them useless in mastication. For many useful teeth are thus affected, and are always easily relieved, and often entirely cured by any highly stimulating application to them and the gums. To brush the teeth and gums frequently every day with a stiff brush, is very advantageous, and, perhaps, more generally efficient than any other remedy.

Exostosis, is another disease of the teeth, which occasions the most intense and excruciating paroxysms; but as the pain is not generally felt in the tooth, nor its paroximate parts, it cannot, with propriety, be considered under the head of tooth-ache, and may, therefore, form the subject of some future paper.

